# Influence of coronary territory on flow profiles of saphenous vein grafts

**DOI:** 10.1186/s13019-018-0709-6

**Published:** 2018-02-20

**Authors:** Sanaz Amin, Raphael S. Werner, Per Lav Madsen, George Krasopoulos, David P. Taggart

**Affiliations:** 1 University of Oxford, Oxford, UK; 2 Department of Cardiovascular Surgery, Oxford University Hospitals Trust, Oxford, UK; 3 Department of thoracic surgery, Faculty of Medicine, University of Zurich, Zurich, Switzerland; 4 Department of Cardiology, Copenhagen University Hospital, Herlev, Denmark

**Keywords:** Transit time flowmetry, TTFM, Coronary artery by-pass surgery, CABG, Intraoperative graft patency assessment, Saphenous vein graft

## Abstract

**Background:**

Differing perfusion of the left and right ventricular coronary territory may influence flow-profiles of saphenous vein grafts (SVGs). We compared flow parameters, measured by transit-time flowmetry (TTFM), in left- and right-sided SVGs during coronary artery by-pass grafting (CABG).

**Methods:**

Routine TTFM measurements were obtained in 167 SVGs to the left territory (55%) and 134 SVGs to the right territory (total of 301 SVGs in 207 patients). The four standard TTFM parameters, [mean graft flow (MGF), pulsatility index (PI), percentage diastolic filling (%DF), and percentage backward flow (%BF)] were compared. Differences in flow parameters were also examined according to surgical technique (on- vs. off-pump).

**Results:**

No significant difference between coronary territories was found for MGF, PI and %BF. However, a higher %DF was noted in left-sided SVGs in the overall cohort as well as in the on-pump (both *p* < 0.001) and the off-pump cohorts (*p* = 0.07). Further, a significantly higher %BF was found in SVGs performed off-pump to the left territory (1.2 ± 2.5 vs. 2.3 ± 3.0, *p* = 0.023). In a multivariate regression analysis, anastomosing a SVG to the left territory was weakly associated with higher PI (*OR* = 0.36, *p* = 0.026) and strongly associated with higher %DF (*OR* = 5.1, *p* < 0.001). No significant association was found for MGF, PI, %DF or %BF in either the on-pump nor the off-pump cohorts.

**Conclusions:**

Although statistically significant, the established differences in TTFM parameters between left- and right-sided vein grafts were small and unlikely to be of clinical relevance.

## Background

Coronary artery by-pass surgery (CABG) remains the optimal treatment for complex coronary artery disease [1], but despite the survival benefit of CABG, long-term graft patency remains a concern. Current European guidelines on CABG recommend intraoperative graft assessment with specific cut-off values for mean graft flow (MGF) and pulsatility index (PI) by transit-time flowmetry (TTFM) technique [2]. However, a limitation of current guideline recommendations is that they do not take into account the known differences in physiological pattern of flow between the right and left coronary artery territories [2].

The transmyocardial pressure is higher on the left side of the heart compared to the right, as the left ventricle provides circulatory support for the high-pressure systemic circulation, and hence a larger fraction of the myocardial blood flow in the left coronary territory takes place during ventricular diastole [3–5]. These differences in coronary blood flow of the left and right coronary territory might therefore be expected to influence flow in by-pass grafts. Few studies, however, have specifically addressed flow in left- vs. right-sided grafts, and have included cohorts of both arterial and venous conduits. Hence, current evidence for any potentially clinically relevant difference is scarce and contradictory [6–8].

The aim of this study was to compare all TTFM parameters MGF, PI, percentage of diastolic filling (%DF), and percentage of backward flow (%BF) in saphenous vein grafts (SVGs) supplying the left and right coronary territories in a larger cohort of CABG patients. Results were then analysed according to on-pump (ONCABG) vs. off-pump (OPCABG). We hypothesized that the higher pressure on the left side would particularly influence diastolic run off in SVGs.

## Methods

### Study design and population

**Fig. 1:**
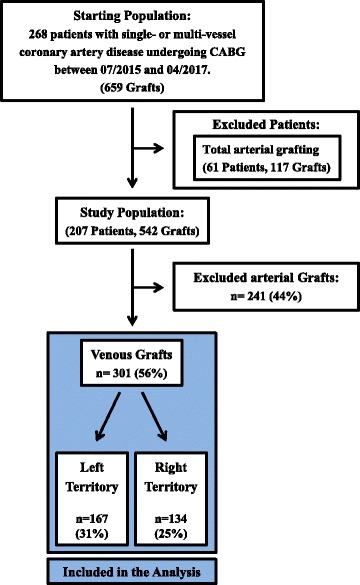
Consort diagram showing exclusion criteria and study group breakdown

*This study was designed as a comparative, non-interventional study, in which data was collected retrospectively*. The population consisted of 268 consecutive CABG patients (total of 659 by-pass grafts) undergoing standard elective or urgent isolated OPCABG or ONCABG. After exclusion of arterial conduits, the study population included 207 CABG patients with a total of 301 SVGs operated in one Centre (John Radcliffe Hospital, Oxford, UK) from July 2015 to April 2017. The study group breakdown is depicted in Fig. 1. TTFM parameters, mean arterial pressure (MAP), demographic data, and risk profile were prospectively collected.

The degree of proximal stenosis in the coronary arteries was assessed by one blinded operator (SA) using quantitative coronary angiography (Horizon Cardiology version 12.2, McKesson, Israel). All TTFM measurements were performed with the VeriQC device (Medistim ASA, Oslo, Norway).

### Procedure

#### General

All patients underwent CABG via a median sternotomy. SVGs were endoscopically harvested as skeletonized conduits and stored in heparinized blood prior to performing the anastomosis. The vast majority of SVGs were used as single grafts to graft the obtuse marginal (OM), the right coronary artery (RCA), or the posterior descending arteries (PDA). However, most grafts anastomosed to the right territory were anastomosed to the PDA. Two surgeons (DPT and GK) performed all CABGs. The routine practice of one surgeon (DPT) was OPCABG, and the routine practice of the other surgeon (GK) was ONCABG.

*The average vessel diameter and the extent of native coronary artery vessel disease was assessed by one blinded operator (SA) using quantitative coronary angiography (QCA) (Horizon Cardiology version 12.2, McKesson, Israel).*

#### Principle of TTFM measurement

Prior to TTFM measurement, the surgeon selected an appropriately sized TTFM probe according to a visual estimation of the external conduit diameter. Mainly probe size 4 or 5 mm was used. Ultrasound gel was applied to the lumen of the probe so that the graft occupied a minimum of 75% of the lumen. During TTFM measurement, the ultrasound probe was placed as close as possible to the distal anastomosis *for the most accurate reading of the flow dynamics across the anastomosis, as previous studies have demonstrated slightly lower %DF and higher PI in the proximal compared to the distal segment of a by-pass graft* [9]*.* TTFM measurements are routinely measured at a mean systemic blood pressure of 75–85 mmHg to exclude the effects on flow of excessively low or high blood pressure. Traction on the pericardium was released, and the stabilizer was removed from the pericardial surface to allow the heart to return to its natural anatomic position. *All TTFM parameters and MAPs were measured after protamine administration. The rational for measuring TTFM after reversal of heparin was to ascertain that the TTFM reading reflected the most accurate interpretation of the graft quality prior to sending the patient out of the operating room.*

#### OPCABG

Complete anticoagulation with heparin was achieved as in the ONCABG group. The lateral and inferior walls were exposed by means of a combination of a deep pericardial stay suture, Trendelenburg and right decubitus position, and opening of the right side of the pericardium to the inferior vena cava. Regional myocardial immobilization was achieved with a suction stabilizer (Octopus Evolution AS, Tissue Stabilizer TS2500, Medtronic Inc.). The target coronary vessels were snared proximally with silastic slings. An intracoronary shunt (Clearview 31,175, Medtronic Inc.) was used during construction of the anastomosis. Shunts were used routinely in all OPCABG cases. A surgical blower-mister device was used to enhance visualization (Clearview 22,150, Medtronic Inc). Proximal anastomoses were made to the ascending aorta at a controlled systolic pressure of between 70 and 80 mmHg and a side-biting vascular clamp.

#### ONCABG

Cardiopulmonary bypass was instituted by ascending aorta cannulation and a two-stage venous cannula in the right atrium. A standard cardiopulmonary by-pass circuit incorporated a roller pump (Jostra HL 20) and a hollow-fiber membrane oxygenator (Inspire Sorin, Fusion Medtronic). The extracorporeal circuit was primed with 1000 mL of Hartmann solution, 500 ml Gelespan, 100 ml mannitol (20% Baxter solution), and 2500 IU of heparin. Non-pulsatile flow was maintained with a flow rate of 2.4 l/min/m^2^. The extra-corporeal circuit was without arterial filtration, and cardiotomy suction was used routinely. Acid-base balance was managed with alpha-stat control. During construction of anastomoses, the patient temperature was allowed to drift to 34 °C before rewarming. Myocardial protection was achieved with intermittent antegrade cold blood-based cardioplegia solution (a mixture of patient’s blood and Harefield cardioplegia solution at ratio of 4:1 during induction and 8:1 for maintenance doses). On completion of all distal anastomoses, the aortic cross-clamp was removed, and the proximal anastomosis was performed with partial clamping.

#### Statistical analysis

Continuous variables are reported as mean ± standard deviation. Normality was assessed with the Kolmogorov-Smirnov test. Comparisons were made with the unpaired t-test for normal distributions and the Mann-Whitney U-test for non-normal distributions. Categorical variables are expressed as frequencies and percentages and were compared using the Chi^2^-Pearson test. A multivariable linear regression model was applied to investigate the effect of the grafted territory (left or right) on TTFM parameters whilst controlling for potential confounding variables. Variables were incorporated into the multivariable analysis if associated with the dependent variables in a univariable analysis (*p* < 0.1). Significance of the multivariable regression model was assessed using the *F*-test. Results of the regression model are presented as regression coefficients (*B*) with 95% confidence intervals (95% CI) and the corresponding *p*-values. A p-value < 0.05 (two-sided) was considered statistically significant. Data analysis was performed in IBM SPSS Statistics Version 22 (SPSS Inc., Chicago, IL).

Statistical power calculations were based on pilot data on MGF from our group and the assumption that for any territory-related difference to be of clinical importance, it should change a normal value by > 10% [10]. With a double-sided alpha-value of 0.05 and a beta-value of 0.80, changing MGF from 48 ml/min by at least 5 ml/min (estimated sigma of 10 ml/min) requires 63 patients in each group. To provide the possibility of further exploration of data including multivariate analysis, we aimed for at least 130 SVGs in each group.

## Results

### Patient baseline characteristics including target vessel disease

Two hundred seven patients receiving a total of 301 SVGs were included in the final analysis (Table 1). 167 SVGs (55%) were grafted to the left territory, and 134 SVGs were grafted to the right territory (Fig. 1). One hundred twenty one patients were operated ONCABG (184 SVGs, 61%), and 86 patients were operated OPCABG (117 SVGs). Patient demographics and their pre-operative risk profile are presented in Table 1. The distribution of the target vessels grafted is shown in Table 2.

**Table 1:** Patient demographics and preoperative risk profile for both groups

Variable	Study population
Total patients	207 (100%)
Age (yrs)	67 ± 8
Male	176 (85%)
Body Mass Index (kg/m^2^)	28.7 ± 4.0
Diabetes mellitus	
Insulin dependent	9 (4%)
Noninsulin dependent	61 (29%)
No history of diabetes	137 (66%)
Chronic obstructive pulmonary disease	24 (12%)
Peripheral vascular disease	22 (11%)
New York Heart Association class	
0	2 (1%)
I	48 (23%)
II	115 (56%)
III	40 (19%)
IV	2 (1%)
Canadian Cardiovascular Society class	
0	13 (6%)
I	19 (9%)
II	132 (64%)
III	32 (15%)
IV	11 (5%)
Left ventricular ejection fraction (%)	58 ± 7
By-pass time (min)	96.1 ± 26.0
Cross-clamp time (min)	66.8 ± 20.8
Surgeon	
”DT”	176 (85%)
"GK”	31 (15%)
Total grafts and territory	542 (100%)
Venous	301 (56%)
Left territory	167 (31%)
Right territory	134 (25%)

**Table 2:** Distribution of target vessels grafted using saphenous vein grafts (SVGs)

Conduit (No.)	LAD	DIAG	IM	OM	RCA	PDA
SVG (301)	5	35	12	115	38	96

*SVG* saphenous vein graft, *LAD* left anterior descending artery, *DIAG* diagonal artery, *IM* intermediate artery, *OM* obtuse marginal branch of circumflex artery, *RCA* right coronary artery, *PDA* posterior descending artery

The extent of native coronary artery vessel disease is shown in Table 3. Target vessel stenosis severity, as judged by QCA, was significant lower in the left coronary territory compared to the right coronary territory both in the overall cohort (86.1 ± 11.8% vs. 90.6 ± 9.8%, *p* < 0.001) and in the OPCABG cohorts (84.5 ± 13 vs. 91.7 ± 9.4%, *p* < 0.04) and ONCABG (87.2 ± 10.4% vs. 90.4 ± 10.1%, *p* = 0.002) cohorts. Non-obstructed target coronary artery diameter, average lumen diameter, and lesion length were comparable between coronary territories.

**Table 3:** Native vessel disease: quantitative coronary angiography data

Variable	Host coronary artery diameter (mm)	Average lumen stenosis diameter (mm)	Host coronary artery stenosis (% area)	Lesion length (mm)
Venous grafts				
Overall (*n* = 301)				
Left territory (*n* = 167)	2.0 ± 0.7	0.69 ± 0.37	86.1 ± 11.8	9.4 ± 4.7
Right territory (*n* = 134)	2.0 ± 0.8	0.67 ± 0.40	90.6 ± 9.8	9.3 ± 4.3
*p*-value	0.9	0.8	< 0.001	0.9
On-pump CABG (*n* = 184)				
Left territory (*n* = 99)	2.0 ± 0.7	0.67 ± 0.36	87.2 ± 10.4	9.6 ± 4.4
Right territory (*n* = 85)	2.0 ± 0.9	0.69 ± 0.44	90.4 ± 10.1	9.0 ± 4.2
*p*-value	0.5	0.4	0.002	0.5
Off-pump CABG (*n* = 117)				
Left territory (*n* = 68)	2.0 ± 0.7	0.71 ± 0.38	84.5 ± 13.5	9.2 ± 5.1
Right territory (*n* = 49)	1.9 ± 0.5	0.65 ± 0.33	91.7 ± 9.4	9.9 ± 4.5
*p*-value	0.6	0.4	0.040	0.4
*p*-value*	0.8	0.5	0.1	0.6
*p*-value**	0.5	0.6	0.5	0.3

*; comparing on- and off-pump CABG to the left territoty

**; comparing on- and off-pump CABG to the right territory

### Comparison of TTFM parameters between left and right coronary territory

**Fig. 2:**
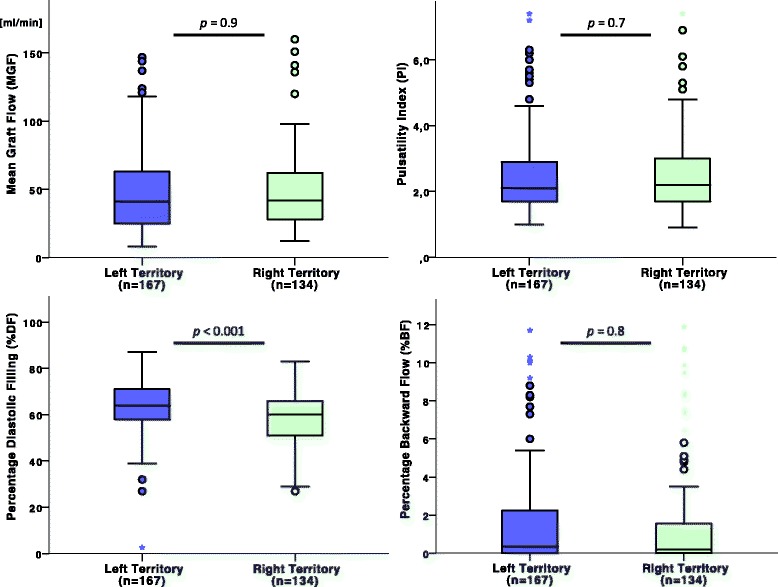
Boxplots depicting differences in mean graft flow (MGF), pulsatility index (PI), percentage diastolic filling (%BF) and percentage backward flow (%BF) between grafts going to the left and right coronary territory

TTFM measurements, MAP at the time of measurement, and by-pass times are presented in Fig. 2 and Table 4. As intended, MAP at the time of TTFM measurement was similar between grafts anastomosed to the left and the right territories. No significant differences between SVGs supplying the two coronary territories were found for MGF, PI, and %BF. However, a higher %DF was noted in left-sided SVGs in comparison to right-sided SVGs in the overall (63.3 ± 12.0% vs. 58.1 ± 10.5%, *p* < 0.001), OPCABG (63.1 ± 12.1% vs. 59.5 ± 10.4%, *p* = 0.07) and ONCABG (63.5 ± 12.0% vs. 57.3 ± 10.5%, *p* < 0.001) cohorts.

**Table 4:** Comparison of intraoperative TTFM parameters between venous grafts going to the left and to the right territory

Variable	Mean graft flow (ml/min)	Pulsatility Index	Diastolic filling (%)	Backward flow (%)	By-pass time (min)	Mean arterial pressure at TTFM (mmHg)
Saphenous vein grafts						
Overall (*n* = 301)						
Left territory (*n* = 167)	48.3 ± 33.8	2.6 ± 1.7	63.3 ± 12.0	1.8 ± 3.2	94.5 ± 23.4	80.9 ± 8.1
Right territory (*n* = 134)	48.3 ± 29.1	2.5 ± 1.2	58.1 ± 10.5	1.6 ± 3.0	98.8 ± 28.4	80.7 ± 6.2
*p*-value	0.9	0.7	< 0.001	0.8	0.3	0.9
On-pump CABG (*n* = 184)						
Left territory (*n* = 99)	51.7 ± 28.1	2.5 ± 1.9	63.5 ± 12.0	1.2 ± 2.5	94.5 ± 23.4	80.8 ± 5.8
Right territory (*n* = 85)	49.0 ± 30.1	2.5 ± 1.3	57.3 ± 10.5	1.6 ± 3.2	98.8 ± 28.4	80.1 ± 5.4
*p*-value	0.5	0.9	< 0.001	0.4	0.3	0.3
Off-pump CABG (*n* = 117)						
Left territory (*n* = 68)	43.5 ± 40.4	2.7 ± 1.4	63.1 ± 12.1	2.3 ± 3.0	–	81.0 ± 10.6
Right territory (*n* = 49)	47.0 ± 27.5	2.6 ± 1.2	59.5 ± 10.4	1.6 ± 2.6	–	82.0 ± 7.3
*p*-value	0.6	0.5	0.071	0.2	–	0.6
*p*-value*	0.1	0.3	0.8	0.023	–	0.9
*p*-value**	0.7	0.7	0.3	0.9	–	0.095

*comparing on- and off-pump CABG to the left territoty

**comparing on- and off-pump CABG to the right territory

### Impact of OPCABG vs. ONCABG on TTFM parameters in the left and right territory

No significant territory-related difference was found for MGF, PI, and %DF between OPCABG and ONCABG cohorts. However, for the left territory, a significantly higher %BF was found in OPCABG SVGs compared to ONCABG SVGs (2.3 ± 3.0% vs. 1.2 ± 2.5%, *p* = 0.023) (Table 4).

### Multivariable linear regression model

The variables MGF, PI, %DF, %BF, mean arterial pressure at TTFM, age, sex, diabetes, surgeon, ONCABG and OPCABG, grafted territory, native coronary artery stenosis and minimal luminal diameter were incorporated into the multivariable analysis based on their association in the univariable analysis (*p* < 0.1). The grafted coronary territory did not have a significant influence on MGF. However, the coronary territory had a significant influence on PI and %DF: Thus, grafting to the left territory led to higher PI and %DF of SVGs (*B* = 0.36, 95% CI 0.04–0.67, *p* = 0.026 and *B* = 5.1, 95% CI 2.86–7.34, *p* < 0.001, respectively). No significant influence of grafted territory was found for %BF. Moreover, no significant association was found between TTFM parameters and OPCABG *or* ONCABG technique.

Some of the covariates used to adjust for the effect of the grafted territory were also independently associated with the TTFM parameters: older age was associated with higher MGF (*p* = 0.034) and lower %DF (*p* < 0.05), and male gender was associated with higher %DF (*p* = 0.025). Furthermore, higher MGF was associated with lower %BF (*p* = 0.012) and greater %DF (*p* = 0.009), while higher PI was associated with greater %BF (*p* < 0.001) and lower %DF (*p* < 0.001). Moreover, higher %DF itself was associated with lower %BF (*p* = 0.004).

## Discussion

As demonstrated by Transit-time flowmetry in patients undergoing CABG, our study has shown that saphenous vein grafts (SVGs) anastomosed to the left and right coronary territory have comparable mean graft flow (MGF), pulsatility index (PI), and percentage of backward flow (%BF) irrespective of surgical technique (OPCABG *or* ONCABG). However, SVGs anastomosed to the left territory have a significantly higher diastolic filling (%DF) than SVGs anastomosed to the right territory both during OPCABG and ONCABG. Moreover, compared to ONCABG, a significantly higher %BF was found in OPCABG SVGs supplying the left coronary territory (Table 4). In a multi-regression analysis, grafting to the left territory was weakly associated with higher PI and strongly associated with higher %DF of SVGs.

In keeping with our finding of no significant difference between territories with respect to MGF, the study by Tokuda et al. [6], including a mixture of arterial and venous grafts, found no statistically significant difference in MGF between grafts anastomosed to the left or the right coronary territory [6]. On the other hand, the study by Kim et al. [7], including a total of 117 arterial conduits (all operated as OPCABG), found a higher MGF in grafts anastomosed to the left than to the right territory. However, as only arterial grafts were assessed, the structural and physiological differences between arterial and venous conduits [11, 12] may explain these conflicting results, and further research is thus warranted to address this topic.

In further concordance with our results of no significant territory-related difference in PI, the study by Tokuda et al. [6] also found no statistically significant difference between grafts anastomosed to the left or the right territory with respect to PI [6]. In contrast to this finding, the study by Kim et al. [7] found a higher PI in grafts anastomosed to the right coronary territory. Again, since the study by Kim et al. [7] only included arterial conduits, this may well explain these conflicting results. Another explanation for why a higher PI may be found in right-sided grafts may be due to the position of the TTFM probe during measurement. Repositioning of the heart into anatomical position at the end of surgery will obscure the distal end of the graft. Consequently, TTFM measurement is often performed proximally on right-sided grafts, where PI is usually higher, *as the high pressured forward flow from the aorta results in higher systolic peak flows in the proximal segment of a by-pass graft and hence in a bigger difference between maximum flow and minimum flow* [9]*, as further explained in the following.* In theory, a slightly higher PI may well be expected in grafts anastomosed to the left territory, as the PI is obtained by dividing the difference between maximum and minimum flow by the mean flow: $\frac{\Big({Q}_{Max}-{Q}_{\mathit{\operatorname{Min}}\Big)}}{Q_{Mean}}$. Therefore, a larger difference between maximum and minimum flow, resulting in a higher PI value, may be expected on the left side of the heart where the higher transmyocardial pressure results in a higher pressure amplitude between systole and diastole [13]. Indeed, the multi-regression analysis suggested that grafting to the left territory was weakly associated with higher PI in SVGs.

In agreement with our study suggesting Tokuda et al. [6] and Kim et al. [7] both demonstrated a higher %DF in grafts anastomosed to the left compared to the right coronary territory compared. As previously noted, a higher %DF is indeed expected on the left side of the heart due to the higher transmyocardial pressure, which lowers systolic coronary flow, irrespective of conduit type (arterial or venous) or surgical technique. However, it is important to acknowledge that the established differences in %DF found in our study is of a mere 5%-points. Although this may reach statistical significance, the established difference is in most situations clinically insignificant.

Tokuda et al. [6] also found no significant difference in %BF in left vs. right-sided by-pass grafts. In contrast, however, Kim et al. [7] found a higher %BF in grafts anastomosed to the left coronary territory. Notably, all grafts were performed by OPCABG technique, which is consistent with our results showing higher %BF only in OPCABG SVGs anastomosed to the left coronary territory (Table 4). A higher %BF is indeed expected on the left side due to the higher transmyocardial pressure, which forces blood backward during isovolumetric contraction. However, these differences were also too small to be of any clinical relevance.

No other parameters were significantly different in grafts anastomosed in OPCABG vs. ONCABG surgery. Notwithstanding this however, previously published papers on TTFM parameters in OPCABG vs. ONCABG found a higher MGF in grafts performed with the latter technique [14–16]. In a prospective study [14] including a total of 266 grafts (203 OPCABG vs. 63 ONCABG) in 100 patients, Taggart and colleagues reported a lower MGF in SVGs performed by OPCABG technique despite a higher mean arterial pressure when compared to ONCABG (*p* < 0.05) [14]. The authors suggested that these findings might be related to vasodilatation following a period of myocardial ischaemia. Indeed, it has been well documented that despite the use of cardioplegia, cross-clamping in ONCABG leads to global myocardial ischaemia and subsequent acidosis with resultant dilatation of coronary arteries [17]. Thus, a higher MGF may be expected in ONCABG vs. OPCABG by-pass grafts. Although our results did demonstrate a numerically higher MGF in the ONCABG SVG cohort, this difference did not reach clinical or statistical significance.

Of note, the multi-regression analysis of this study showed significant associations between the four TTFM parameters, which suggests that these parameters should thus be considered complementary rather than in isolation when assessing quality of SVGs intraoperatively [4, 5, 10]. This is supported by studies demonstrating that high quality grafts, as determined by angiographic graft patency assessment, have high MGF due to good run-off with concomitant low PI, predominant %DF, and little %BF [6, 18–22].

## Conclusions

Saphenous vein grafts supplying the left coronary artery territory have a higher diastolic flow (%DF) when compared to right-sided SVGs, irrespective of whether the procedure is performed ONCABG or OPCABG. Furthermore, multi-regression analysis suggests that SVGs grafted to the left coronary territory are weakly associated with higher PI. The magnitude of differences are numerically small and unlikely to be of clinical significance.

### Limitations

Our study was observational and confounding cannot be excluded due to lack of randomisation. Further, while the statistical power-analysis allowed for clinically significant changes to be excluded, we cannot exclude if smaller changes may be of importance for long-term patency. Furthermore, the extent of host coronary artery stenosis was statistically higher in the right coronary territory (Table 3). However, the mean host coronary artery stenosis was over 80% in both territories, which should make the difference between the two territories clinically irrelevant and results thus comparable [23]. Notably, this study only describes perioperative flow-profiles of SVGs; hence these results may not necessarily reflect the long-term flow-profile in either group.

## Abbreviations

BF: Backward flow
CABG: Coronary artery bypass grafting
DF: Diastollic filling
MGF: Mean graft flow
OM: Obtuse marginal artery
ONCABG: On-pump coronary artery bypass grafting
OPCABG: Off-pump coronary artery bypass grafting
PDA: Posterior descending artery
PI: Pulsatility index
RCA: Right coronary artery
SVG: Saphenous vein graft
TTFM: Tansit time flowmetry
